# Isolated unilateral sixth nerve palsy in a patient with nasopharyngeal carcinoma

**Published:** 2019

**Authors:** Kaveh Abri Aghdam, Amin Zand, Mostafa Soltan Sanjari

**Affiliations:** * Eye Research Center, The Five Senses Institute, Rassoul Akram Hospital, Iran University of Medical Sciences, Tehran, Iran

**Keywords:** sixth nerve palsy, nasopharyngeal carcinoma, horizontal diplopia, nasopharynx

## Abstract

**Objective:** To describe an isolated unilateral sixth nerve palsy, as a rare neuro-ophthalmic presentation of nasopharyngeal carcinoma.

**Methods:** We report a 54-year-old female, known case of nasopharyngeal carcinoma who was treated with chemo-radiotherapy, and presented with isolated right sixth nerve palsy. Magnetic resonance imaging (MRI) indicated extension of tumor to intracranial fossa with clival involvement.

**Results:** The patient was referred to an otolaryngologist for further evaluation and necessary intervention due to invasion of the cancer to intracranial fossa with involvement of right abducens nerve.

**Conclusions:** Although, isolated sixth nerve palsies in adults over the age of 50 are usually ischemic; but in enduring cases, neoplastic processes should be considered.

## Introduction

The nasopharynx is anatomically situated precisely beneath the pedestal of the skull. Nasopharyngeal carcinoma is an aggressive tumor which is hard to control. Common clinical exhibitions are cervical masses, bloody nasal discharge, ear symptoms and cranial nerve palsy. When this carcinoma presents with cranial nerve involvement, the prognosis of the patient will be poor. In patients suffering from cranial nerve palsies, fifth and sixth nerves are the most frequently involved. However, an isolated unilateral sixth nerve palsy is a rare symptom of nasopharyngeal carcinoma in the skull base and few such cases have been reported in the literature so far.

## Materials and methods - Case Report

The patient was a 54-year-old female with prior history of invasive nasopharyngeal carcinoma from 7 months ago who was treated with chemo-radiotherapy. She complained of dizziness and occasional horizontal diplopia four months previous to presentation. She had lost weight significantly over this period. The patient did not have any symptoms or signs associated with the disease such as epistaxis, reduced hearing, tinnitus or hyposmia. She had no history of smoking or any systemic disease like diabetes mellitus or hypertension. The patient reported no obvious headache, visual loss or jaw claudication recently.

**Fig. 1:**
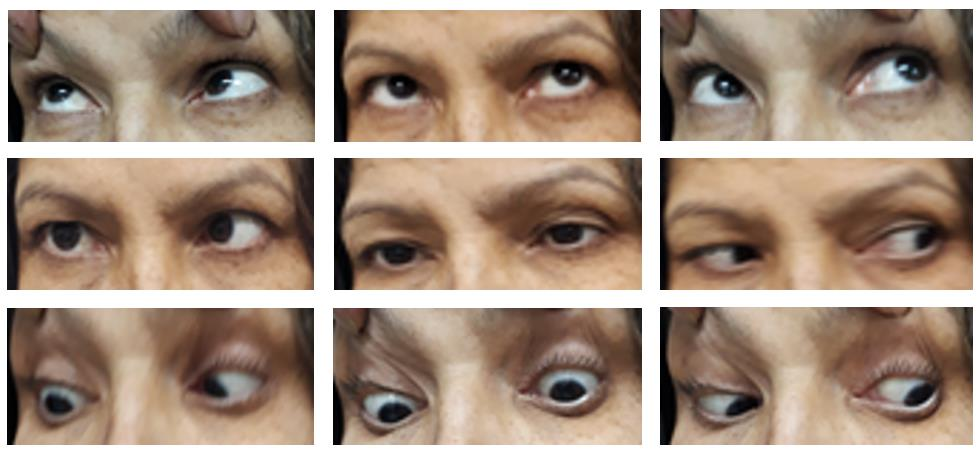
Right abduction deficit, prominent on right lateral gaze

**Fig. 2:**
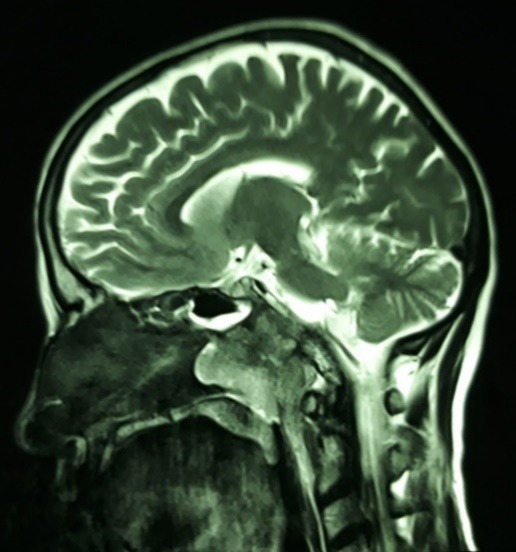
Brain and cervical MRI, T2 weighted, sagittal cut: heterogeneously enhancing mass in the right aspect of the nasopharynx

**Fig. 3:**
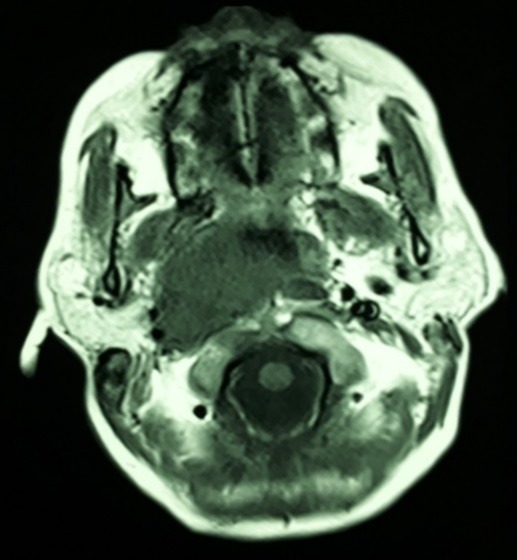
Brain MRI, T2 weighted, axial cut: upward extension of the heterogeneous mass into the middle cranial fossa, that involved the clivus

Ophthalmic examination exhibited a best-corrected visual acuity of 10/10 in both eyes (by Snellen E letter chart, at a distance of six meters). Her pupillary reactions to light and accommodation were normal in both eyes, and relative afferent pupillary defect was absent. The patient had right face turn with an abduction deficit of the right eye (about 3-) (**Fig. 1**). Ocular motility was within normal limits in the left eye. Alternate cover testing revealed right esotropia in primary position, which was greatest in the right gaze. Intraocular pressures were inside normal range in both eyes by applanation tonometry. Color plate testing results (by Ishihara's color plate test) for the both eyes were 14/14. Slit-lamp examination was unremarkable. Dilated fundus examination of both eyes was within normal bounds without any optic disc abnormalities like papilledema or infiltrative optic neuropathy. Cervical and brain MRI showed a bulky multifarious emerging mass in the right aspect of the nasopharynx (**Fig. 2**) that extended to the middle cranial fossa with clival involvement (**Fig. 3**). The patient was referred to an otolaryngologist for further evaluation and necessary intervention due to infiltration of the cancer to intracranial fossa with engagement of right abducens nerve.

## Discussions

Nasopharyngeal carcinoma is an intrusive tumor with a rare incidence about one per 100,000. Due to superior enlargement of the tumor, cranial nerve engagement can ensue. Invasion of nasopharyngeal carcinoma into the cavernous sinus could affect cranial nerves III, IV, V, and VI. The optic nerve can be engaged in cases with parasellar invasion of the tumor [**1**].

The most commonly involved cranial nerves are nerve V (particularly maxillary division) and VI [**1**-**4**]. Sixth nerve palsy instigates lateral rectus muscle paralysis that produces diplopia and abduction deficit of the involved eye. While paralysis of the fifth nerve can lead to hypoesthesia of the portions of the face depended to the location of involvement. Interestingly, our patient developed an isolated sixth nerve palsy, as an unusual neuro-ophthalmic presentation for this tumor [**5**,**6**]. She had upward growth of the tumor into the middle cranial fossa with engagement of the clivus, that led to right sixth neve palsy with the presentation of abduction deficit of the right eye with occasionally horizontal diplopia.

The causes of fresh-start diplopia in patients with nasopharyngeal carcinoma are tumor recurrence or complications of radiotherapy. The diagnosis of radiation-induced cranial nerve palsy is usually made by exclusion of tumor recurrence as the initiator of neuropathy [**7**].

Cranial nerves palsy (especially third, fourth and sixth nerves) as the presenting symptom of nasopharyngeal carcinoma recurrence are common with an incidence rate of 20-38% [**8**,**9**]. In a study by Kau HC et al., the majority of the new onset diplopia in post-treated nasopharyngeal carcinoma patients was a consequence of tumor recurrence (52%) [**7**]. In post-treated patients with nasopharyngeal carcinoma, imaging techniques like computed tomography (CT) scanning and magnetic resonance (MR) imaging are necessary and useful tools for evaluation of tumor recurrence. The common imaging findings in patients with nasopharyngeal carcinoma are destruction of the skull pedestal and intracranial growth into the middle cranial fossa. In cases with cranial nerve palsy indicating intracranial extension of the tumor, destruction of the skull base is the most typical finding [**10**,**11**], as the upward enlargement of the tumor to intracranial fossa is apparent in the brain MRI. 

## Results

Due to invasion of cancer into the intracranial fossa with the involvement of right abducens nerve, we referred the patient to an otolaryngologist for further evaluation and to rule out tumor recurrence.

## Conclusions

Isolated sixth nerve palsies in adults over the age of 50 are usually ischemic, but in enduring cases, neoplastic processes should be considered. This entails medical and paramedical (especially brain and orbital imaging) evaluation.

Also, new onset diplopia or any symptoms of cranial nerve palsy in post-treated nasopharyngeal carcinoma patients need further evaluation for exclusion of tumor recurrence. In this situation, performing a detailed clinical history and using imaging modalities are necessary. 
